# Assessment of Tissue Expression of the Oxytocin–Vasopressin Pathway in the Placenta of Women with a First-Episode Psychosis during Pregnancy

**DOI:** 10.3390/ijms241210254

**Published:** 2023-06-16

**Authors:** Miguel A. Ortega, Cielo García-Montero, Óscar Fraile-Martinez, Diego De Leon-Oliva, Diego Liviu Boaru, Coral Bravo, Juan A. De Leon-Luis, Miguel A. Saez, Angel Asúnsolo, Ignacio Romero-Gerechter, Alejandro Sanz-Giancola, Raul Diaz-Pedrero, Laura Lopez-Gonzalez, Luis G. Guijarro, Silvestra Barrena-Blázquez, Julia Bujan, Natalio García-Honduvilla, Melchor Alvarez-Mon, Miguel Ángel Alvarez-Mon, Guillermo Lahera

**Affiliations:** 1 Department of Medicine and Medical Specialities, Faculty of Medicine and Health Sciences, University of Alcalá, 28801 Alcalá de Henares, Spain; cielo.gmontero@gmail.com (C.G.-M.); oscarfra.7@hotmail.com (Ó.F.-M.); diegodleonoliva01@gmail.com (D.D.L.-O.); diego.boaru@edu.uah.es (D.L.B.); msaega1@oc.mde.es (M.A.S.); mjulia.bujan@uah.es (J.B.); natalio.garcia@uah.es (N.G.-H.); mademons@gmail.com (M.A.-M.); maalvarezdemon@icloud.com (M.Á.A.-M.); guillermo.lahera@gmail.com (G.L.); 2 Ramón y Cajal Institute of Sanitary Research (IRYCIS), 28034 Madrid, Spain; angel.asunsolo@uah.es (A.A.); raul.diazp@uah.es (R.D.-P.); laura.lgonzalez@uah.es (L.L.-G.); luis.gonzalez@uah.es (L.G.G.); silvebarrena@gmail.com (S.B.-B.); 3 Department of Public and Maternal and Child Health, School of Medicine, Complutense University of Madrid, 28040 Madrid, Spain; cbravoarribas@gmail.com (C.B.); jaleon@ucm.es (J.A.D.L.-L.); 4 Department of Obstetrics and Gynecology, University Hospital Gregorio Marañón, 28007 Madrid, Spain; 5 Health Research Institute Gregorio Marañón, 28009 Madrid, Spain; 6 Pathological Anatomy Service, Central University Hospital of Defence-UAH Madrid, 28801 Alcalá de Henares, Spain; 7 Department of Surgery, Medical and Social Sciences, Faculty of Medicine and Health Sciences, University of Alcalá, 28801 Alcalá de Henares, Spain; 8 Psychiatry Service, Center for Biomedical Research in the Mental Health Network, University Hospital Príncipe de Asturias, 28801 Alcalá de Henares, Spain; iromeroger@gmail.com (I.R.-G.); sirgiancola@gmail.com (A.S.-G.); 9 Unit of Biochemistry and Molecular Biology (CIBEREHD), Department of System Biology, University of Alcalá, 28801 Alcalá de Henares, Spain; 10 Department of Nursing and Physiotherapy, Faculty of Medicine and Health Sciences, University of Alcalá, 28801 Alcalá de Henares, Spain; 11 Immune System Diseases-Rheumatology and Internal Medicine Service, University Hospital Príncipe de Asturias, CIBEREHD, 28806 Alcalá de Henares, Spain; 12 Department of Psychiatry and Mental Health, Hospital Universitario Infanta Leonor, 28031 Madrid, Spain

**Keywords:** placenta, oxytocin (OXT), arginine vasopressin (AVP), oxytocin receptor (OXTR), arginine vasopressin receptor 1A (AVPR1a), first-episode psychosis (FEP)

## Abstract

Psychosis refers to a mental health condition characterized by a loss of touch with reality, comprising delusions, hallucinations, disorganized thought, disorganized behavior, catatonia, and negative symptoms. A first-episode psychosis (FEP) is a rare condition that can trigger adverse outcomes both for the mother and newborn. Previously, we demonstrated the existence of histopathological changes in the placenta of pregnant women who suffer an FEP in pregnancy. Altered levels of oxytocin (OXT) and vasopressin (AVP) have been detected in patients who manifested an FEP, whereas abnormal placental expression of these hormones and their receptors (OXTR and AVPR1A) has been proven in different obstetric complications. However, the precise role and expression of these components in the placenta of women after an FEP have not been studied yet. Thus, the purpose of the present study was to analyze the gene and protein expression, using RT-qPCR and immunohistochemistry (IHC), of OXT, OXTR, AVP, and AVPR1a in the placental tissue of pregnant women after an FEP in comparison to pregnant women without any health complication (HC-PW). Our results showed increased gene and protein expression of OXT, AVP, OXTR, and AVPR1A in the placental tissue of pregnant women who suffer an FEP. Therefore, our study suggests that an FEP during pregnancy may be associated with an abnormal paracrine/endocrine activity of the placenta, which can negatively affect the maternofetal wellbeing. Nevertheless, additional research is required to validate our findings and ascertain any potential implications of the observed alterations.

## 1. Introduction

Psychosis is an amalgamation of psychological symptoms resulting in a loss of contact with reality [1]. The *Diagnostic and Statistical Manual of Mental Disorders, 5th Edition* (DSM-V), the principal authority for psychiatric diagnoses in the United States, characterizes psychotic disorders as deviations within one of the five specified domains: delusions, hallucinations, disorganized thought, disorganized behavior (including catatonia), and negative symptoms [2]. Schizophrenia, as the archetypal form of psychotic disorder, is characterized by the enduring presence of psychotic symptoms and concurrent impairment in functioning. However, the manifestation of psychosis extends across a broad spectrum of conditions, encompassing major depressive disorder, bipolar disorder, schizoaffective disorder, as well as delusional and schizophreniform disorder [3]. A systematic review and meta-analysis of 73 articles by Moreno-Küstner et al. describes a global prevalence of 4.6 per 1000 people [4]. Despite the fact that the etiology of psychotic disorders remains poorly understood, it is a complex and multifactorial process that involves a combination of genetic [5,6] and environmental factors [3,7].

First-episode psychosis (FEP) during pregnancy is very unlikely, but it may adversely affect maternofetal wellbeing [8]. Within the context of psychosis during pregnancy, several prominent risk factors have been identified, including a familial history of psychosis, a previous occurrence of psychosis during pregnancy, and the presence of pre-existing or undiagnosed psychotic or mood disorders, such as schizophrenia or bipolar disorder [9]. Prior research has demonstrated that women experiencing a psychotic episode during pregnancy exhibit elevated susceptibility to a range of unfavorable obstetric and neonatal outcomes, including but not limited to cesarean delivery, impaired fetal growth, placental abruption, antepartum or postpartum hemorrhage, fetal anomalies, fetal distress, or stillbirth [9,10]. FEP can lead to long-term consequences affecting the wellbeing of the woman, the newborn, her family, and wider society [11]. Despite this, there is little knowledge about the pathophysiology of FEP, and its management remains challenging for clinicians, who must take into consideration the benefits and harms [12].

To date, the potential role of the placenta in the maternofetal distress exhibited by pregnant women with psychosis has not been well characterized. The placenta is a pivotal and transient organ that sustains the growth of the fetus for nine months. To achieve this, the placenta fulfills pleiotropic functions including maternofetal exchange, endocrine activity, mechanical, chemical, and immunological barrier, and determining maternofetal programming, even beyond gestation [13]. Our previous research has uncovered different structural changes in the placental tissue of women with FEP, such as increased oxidative stress [14], increased lipid peroxidation with evidence of ferroptosis [15], and Tenney–Parker changes related to enhanced hypoxia and apoptosis [16]. These changes in the structure of the placenta may be a consequence of the psychotic process and could lead to an impairment of fetal neurodevelopment [17], so its analysis may lead to protective measures of maternofetal wellbeing.

Hormones originating from the placenta have significant involvement in various gestational processes, encompassing implantation, placentation, vascular remodeling, immunomodulation, breastfeeding, and labor [18,19]. Oxytocin (OXT) and arginine vasopressin (AVP) are two nonapeptides that differ in two amino acids synthesized in the supraoptic (SON) and paraventricular (PVN) nuclei by magnocellular and parvocellular neurons, although AVP also can be released in the suprachiasmatic nucleus [20]. OXT binds to its receptor (OXTR) in maternofetal structures, being pivotal in the control of uterine contractions and parturition [21]. Additionally, both components are expressed in the placenta, amnion, chorion, and decidua [22,23]. Therefore, placental OXT may play a role in labor through a paracrine system. The pivotal functions of AVP encompass the maintenance of fluid balance, regulation of vascular tone, and modulation of the endocrine stress response [24]. AVP can bind to three different AVP receptors that are G-coupled protein receptors (GPCR), which is the same as OXTR, and they are expressed in different cell types: V1A, V1B (or V3), or V2. The placenta expresses different types of AVP receptors such as the AVP receptor 1A (AVP1AR), which is also found in vascular smooth muscle and regulates vasoconstriction and blood pressure [25,26]. Alterations in OXT, AVP and their receptors are related to different obstetric complications and mental disorders related to pregnancy like postpartum depression (PPD) [27,28] or preeclampsia [29,30]. In addition, disruptions in the oxytocinergic and vasopressinergic systems have been previously detected in women suffering an FEP [31,32]. However, the precise role of OXT, AVP, and their receptors OXTR and AVPR1A in the placenta of pregnant women after an FEP remains to be fully elucidated.

Thus, the objective of this study was to determine gene and protein expression of OXT, OXTR, AVP, and AVPR1A in the placental tissue of FEP women, when compared to nonpathological control subjects. To accomplish this objective, both gene and protein expression levels of OXT, OXTR, AVP, and AVPR1A were examined using real-time quantitative PCR (RT-qPCR) and immunohistochemistry techniques, respectively.

## 2. Results

### 2.1. The Placentas of Women Who Suffered a First Episode of Psychosis in Pregnancy Exhibited Increased Gene and Protein Expression of Oxytocin and Its Receptor

First, our results showed increased OXT and OXTR in the placentas of women who underwent a first episode of psychosis during pregnancy.

**Figure 1:**
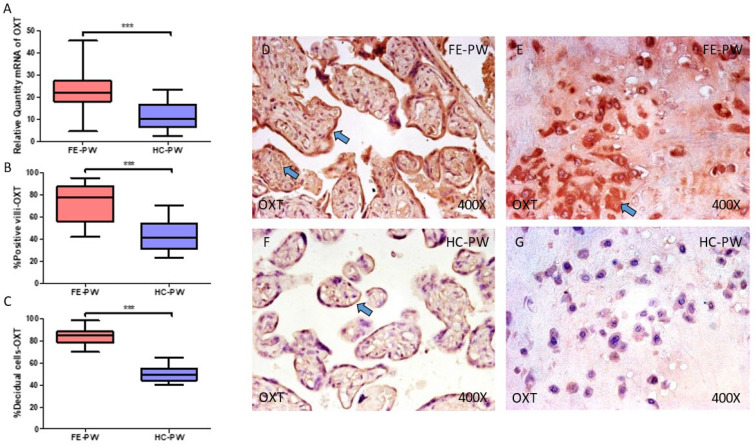
(**A**) Gene expression of oxytocin (OXT) mRNA in the groups of women with first-episode psychosis during pregnancy (FE-PW) and healthy controls (HC-PW). (**B**) IRS scores for OXT expression in the FE-PW group and the HC-PW group’s placental villi. (**C**) IRS scores for OXT expression in the FE-PW group and the HC-PW group’s decidual cells. (**D**,**E**) Photographs demonstrating OXT immunostaining in the placental villi and decidual cells of the FE-PW group, respectively. (**F**,**G**) Photographs demonstrating OXT immunostaining in the placental villi and decidual cells of the HC-PW group, respectively. *p* < 0.001 (***). Blue arrows show increased tissue expression of OXT in the different parts of the placental villi and decidual cells in FE-PW (**D**,**E**), whereas OXT expression is almost limited to syncytiotrophoblast layer in HC-PW (**F**).

Regarding OXT, we observed that gene expression detected using RT-qPCR was significantly higher in FE-PW when compared to HC-PW (FE-PW = 22.063 [4.654–45.351]; HC-PW = 10.482 [2.546–23.352], *** *p* < 0.001, Figure 1A). Immunohistochemical analysis demonstrated that protein expression of OXT was also notably raised in the placental villi of women with FE-PW (FE-PW = 78.000 [42.000–95.000]; HC-PW= 41.000 [23.000–70.000], *** *p* < 0.001, Figure 1B) as well as in decidual cells (FE-PW = 85.000 [70.000–98.000]; HC = 49.000 [40.000–65.000], *** *p* < 0.001, Figure 1C). Histological pictures show protein expression of OXT in the placental villi and decidual cells of FE-PW (Figure 1D,E) and in HC-PW (Figure 1F,G). As can be observed, protein expression of OXT was strongly marked in the different parts of the placental villi of FE-PW, whereas in HC-PW, its expression was almost limited to syncytiotrophoblasts (Figure 1D vs. Figure 1F). In parallel, OXT expression was quite marked in the decidua and decidual cells in FE-PW, and its expression was much less appreciated in the decidual cells (Figure 1E vs. Figure 1G).

**Figure 2:**
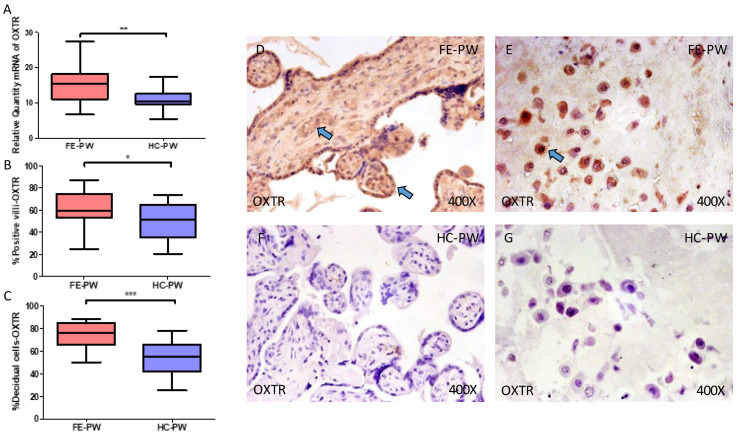
(**A**) Gene expression of oxytocin receptor (OXTR) mRNA in the groups of women with first-episode psychosis during pregnancy (FE-PW) and healthy controls (HC-PW). (**B**) IRS scores for OXTR expression in the FE-PW group and the HC-PW group’s placental villi. (**C**) IRS scores for OXTR expression in the FE-PW group and the HC-PW group’s decidual cells. (**D**,**E**) Photographs demonstrating OXTR immunostaining in the placental villi and decidual cells of the FE-PW group, respectively. (**F**,**G**) Photographs demonstrating OXTR immunostaining in the placental villi and decidual cells of the HC-PW group, respectively. *p* < 0.05 (*); *p* < 0.01 (**); *p* < 0.001 (***). Blue arrows show increased tissue expression of OXTR in the different parts of the placental villi and decidual cells in FE-PW (**D**,**E**), whereas for HC-PW, this expression is less noticeable.

In the case of OXTR, gene expression was also significantly higher in FE-PW when compared to HC (FE-PW = 15.442 [6.655–27.362]; HC = 10.321 [5.362–17.362], ** *p* = 0.0039, Figure 2A). Immunohistochemical analysis demonstrated that protein expression of OXTR was also notably raised in the placental villi of women with FE-PW (FE-PW = 59.500 [25.000–87.000]; HC = 51.500 [20.000–74.000], * *p* = 0.0464, Figure 2B) as well as in decidual cells (FE-PW = 75.500 [50.000–88.000]; HC = 55.000 [25.000–78.000], *** *p* < 0.001, Figure 2C). Histological pictures show protein expression of OXTR in the placental villi and decidual cells of FE-PW (Figure 2D,E) and in HC-PW (Figure 2F,G). More specifically, OXTR is expressed in the placenta of FE-PW throughout the different structures of the placental villi, whereas the brown staining for HC-PW is significantly lower (Figure 2D versus Figure 2F). Similarly, OXTR is notably higher in decidual cells of FE-PW, whereas in HC-PW, OXTR is virtually absent (Figure 2E versus Figure 2G).

### 2.2. The Placentas of Women Who Suffered a First-Episode Psychosis in Pregnancy Displayed Enhanced Gene and Protein Expression of Vasopressin and Vasopressin Type 1a Receptor

On the other hand, we also reported increased expression of AVP and AVPR1A in the placentas of women who underwent a first-episode psychosis during pregnancy.

**Figure 3:**
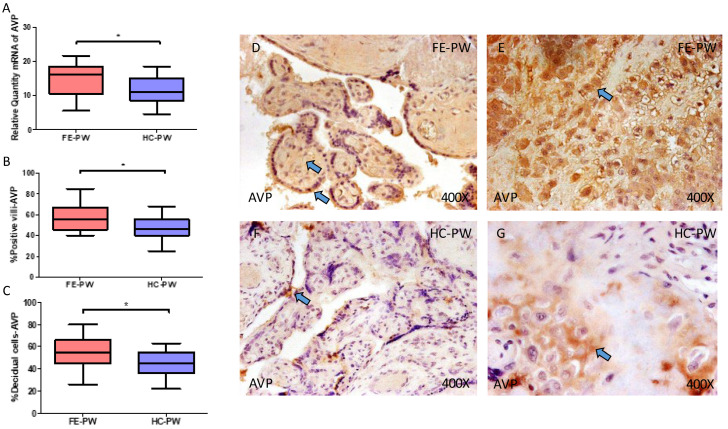
(**A**) Gene expression of arginine vasopressin (AVP) mRNA in the groups of women with first-episode psychosis during pregnancy (FE-PW) and healthy controls (HC-PW). (**B**) IRS scores for AVP expression in the FE-PW group and the HC-PW group’s placental villi. (**C**) IRS scores for AVP expression in the FE-PW group and the HC-PW group’s decidual cells. (**D**,**E**) Photographs demonstrating AVP immunostaining in the placental villi and decidual cells of the FE-PW group, respectively. (**F**,**G**) Photographs demonstrating AVP immunostaining in the placental villi and decidual cells of the HC-PW group, respectively. *p* < 0.05 (*). Blue arrows show increased tissue expression of AVP in the different parts of the placental villi and decidual cells in FE-PW (**D**,**E**), whereas AVP expression is almost limited to the syncytiotrophoblast layer in the placental villi of HC-PW (**F**) as well as in decidual cells, although the level of expression is less marked than FE-PW (**G**).

Regarding AVP, we observed that gene expression detected using RT-qPCR was notably higher in FE-PW when compared to HC (FE-PW = 16.163 [5.562–21.652]; HC = 11.036 [4.561–18.562], * *p* = 0.0162, Figure 3A). The immunohistochemical analysis defined that protein expression of AVP was also significantly elevated in the placental villi of women with FE-PW (FE-PW = 56.000 [40.000–85.000]; HC = 46.000 [25.000–68.000], * *p* = 0.0139, Figure 3B) as well as in decidual cells (FE-PW = 55.000 [26.000–80.000]; HC = 45.000 [22.000–63.000], * *p* = 0.034, Figure 3C). Histological images show protein expression of AVP in the placental villi and decidual cells of FE-PW (Figure 3D,E) and in HC-PW (Figure 3F,G). It can be observed that the placenta of FE-PW showed a marked staining of AVP, especially in the syncytiotrophoblast layer, whereas in the case of HC-PW, protein expression of AVP was significantly reduced (Figure 3D versus Figure 3F). Similar observations can be made in the decidual layer of the placenta in FE-PW when compared to HC-PW (Figure 3E versus Figure 3G).

**Figure 4:**
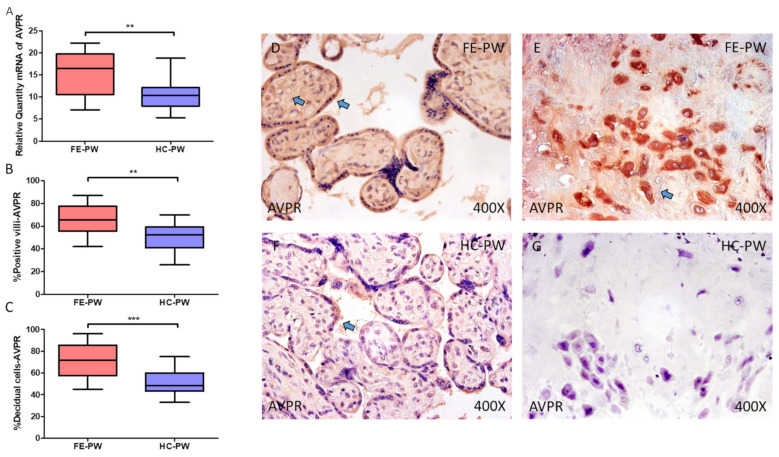
(**A**) Gene expression of arginine vasopressin receptor 1A (AVPR1A) mRNA in the groups of women with first-episode psychosis during pregnancy (FE-PW) and healthy controls (HC-PW). (**B**) IRS scores for AVPR1A expression in the FE-PW group and the HC-PW group’s placental villi. (**C**) IRS scores for AVPR1A expression in the FE-PW group and the HC-PW group’s decidual cells. (**D**,**E**) Photographs demonstrating AVPR1A immunostaining in the placental villi and decidual cells of the FE-PW group, respectively. (**F**,**G**) Photographs demonstrating AVPR1A immunostaining in the placental villi and decidual cells of the HC-PW group, respectively. *p* < 0.01 (**); *p* < 0.001 (***). Blue arrows show increased tissue expression of AVPR1A in the different parts of the placental villi and decidual cells in FE-PW (**D**,**E**), whereas AVPR1A expression is almost limited to the syncytiotrophoblast layer in the placental villi of HC-PW (**F**).

In the case of AVPR1A, gene expression was significantly higher in FE-PW when compared to HC (FE-PW = 16.460 [7.116–22.256]; HC = 10.341 [5.321–18.799], ** *p* = 0.0036, Figure 4A). Immunohistochemical analysis demonstrated that protein expression of AVPR1A was also notably raised in the placental villi of women with FE-PW (FE-PW = 65.500 [42.000–87.000]; HC = 52.500 [26.000–70.000], ** *p* = 0.001, Figure 4B) as well as in decidual cells (FE-PW = 71.500 [45.000–96.000]; HC = 48.500 [33.000–75.000], *** *p* < 0.001, Figure 4C). Histological pictures show protein expression of AVPR1A in the placental villi and decidual cells of FE-PW (Figure 4D,E) and HC-PW (Figure 4F,G). AVPR1A was expressed in the different cells of the placental villi of FE-PW, especially in the syncytiotrophoblast layer, which also seems to express this receptor in HC-PW (Figure 4D versus Figure 4F). In the case of decidual cells, protein expression of AVPR1A can be notably observed in FE-PW, but in HC-PW, its expression seems to be low (Figure 4E versus Figure 4G).

## 3. Discussion

FEP during pregnancy is rare but potentially harmful to maternofetal wellbeing. Previous studies have shown that pregnant women who experience psychotic episodes are more prone to various adverse obstetric and neonatal outcomes [10]. However, the pathogenic mechanisms connecting FEP with adverse obstetric and neonatal outcomes are poorly understood. Previous studies have found that the placentas of FE-PW show evidence of increased cell death, hypoxia, ferroptosis, and oxidative stress [14,15,16], suggesting a possible link between this organ and suffering from FEP during pregnancy. In this work, we have demonstrated abnormal expression of OXT and AVP and their main receptors (OXTR and AVPR1A) in the chorionic villi as well as in the decidual cells of placenta of FEP women during pregnancy, gaining further insights into the pathogenic implications of this condition in the placenta.

OXT and AVP are two neurohypophyseal hormones that are secreted into circulation or within the brain. In the bloodstream, they bind to their receptors on the surface of the plasma membrane to exert their actions. OXT induces contractions of the smooth muscle cells in the myometrium during labor and facilitates the ejection of milk during lactation [33]. AVP manages the regulation of osmotic balance, blood pressure, sodium homeostasis, and kidney function in the body [24]. In addition, OXT and AVP released within the brain are involved in the regulation of social behavior [34,35]. The oxytocinergic and vasopressinergic systems are intimately related, exhibiting promiscuity through the cross-talk among OXTR and AVPR [36]. However, in psychotic disorders the specific contribution of OXTR versus AVPR has not been thoroughly clarified. Previous research has highlighted the involvement of OXT/AVP pathways in psychotic disorders such as schizophrenia [37]. Recently, Hidalgo-Figueroa et al. found that FEP patients present low oxytocin and high prolactin serum levels, poor premorbid IQ, poor sustained attention in women and better-sustained attention in men [31]. Indeed, the administration of exogenous oxytocin (commonly intranasally) has demonstrated benefits in positive symptoms, negative symptoms, and cognitive deficits of schizophrenia [38,39,40]. Studies in animal models suggest that oxytocin may have therapeutic potential by modulating presynaptic dopamine function, thereby addressing the dopamine hyperactivity observed in the mesolimbic pathway [41]. This modulation is believed to contribute to the improvement of positive symptoms in schizophrenia [40]. Additionally, oxytocin appears to counteract the hypoglutamatergic profile associated with the disorder. On the other hand, Rubin et al. found increased serum levels of AVP and were positively correlated with the severity of positive symptoms and impaired cognition in female individuals with untreated FEP, while no such correlation was observed in males [32]. Throughout pregnancy and during the initial and subsequent stages of labor, plasma levels of OXT rise gradually, accompanied by an augmentation in the frequency and size of OXT pulses [42] as well as in the chorion-decidua [43]. At the same time, uterine sensitivity increased due to the upregulation of OXTR in the endometrium, myometrium, chorion-decidua and amnion [43,44]. By contrast, AVP plasma levels remain unchanged during pregnancy, despite the decreased osmolality [45,46]. Likewise, previous studies have also found a potential association between altered OXT and AVP plasma levels with the risk of suffering mental disorders in pregnancy, especially PPD [47,48]. Nonetheless, to our best knowledge, circulating levels of OXT and AVP in FE-PW have not been studied yet, and the pathogenic role of these hormones in these patients remains to be further explored. However, due to the potential association found in previous studies between the OXT/AVP pathway and psychosis, we hypothesized that FEP during pregnancy could be associated with an abnormal expression of this pathway in the placenta, although the directionality and consequences derived from this association are mostly unknown.

First, our findings show a gene and protein overexpression of OXT and OXTR in both placental villi and decidual cells of FE-PW compared to HC. OXT and OXTR play an important role in the activation, stimulation, and involution phases of the parturition [49]. Placental OXT induces uterine contractions by directly affecting the myometrium as well as indirectly by increasing the production of prostaglandins, especially prostaglandin F2 α (PGF2 α), by the decidua, which upregulates OXTR expression and formation of gap junctions that also promote contractions [50]. OXT production and OXTR expression by decidual cells seem to be particularly important during labor, as defended in previous works [51,52]. The pathogenic implications of OXT and OXTR overexpression in FE-PW must be deeply studied. Prior research has found that high levels of oxytocin can increase the intensity of oxidative stress in pregnant women [53]. Additionally, OXT can enhance the effects of hypoxia-inducible factor 1 (HIF-1) signaling [54]. As we previously noticed an increased oxidative stress and hypoxia in the placenta of FE-PW [14,16], it is likely that OXT overexpression might partially contribute to these changes. In terms of OXTR, previous studies have found a differential methylation status of this component in the placenta of women with maternal depression after receiving antidepressants [28], whereas an altered epigenetic status of this receptor in the placenta is associated with insensitivity to OXT in the placental vasculature of women with pre-eclampsia [55]. Hence, it would be interesting to deepen our understanding of the methylation status of OXTR and possible consequences in pregnant women who undergo an FEP, especially considering the therapeutic regimes received.

On the other hand, different factors have been shown to regulate OXT and OXTR. Estrogens, synthesized in the placenta, stimulate the synthesis of OXT in this organ in a paracrine manner [56]. The estrogen hypothesis postulates that estrogen provides a protective effect by mitigating the risk and severity of schizophrenia in females [57]. This fact may partly explain the relatively low frequency of FEP during pregnancy since estrogens are very elevated in this state. Anandamide (AEA) is an endocannabinoid that has been shown to positively regulate the OXT/OXTR system in the placenta [58]. Intriguingly, this component seems to be elevated in the plasma and cerebrospinal fluid of patients with psychosis [59,60,61,62]. It would be interesting to study if both AEA or estrogens could play a possible role in the increase in OXT and OXTR expression in the placenta of FE-PW, although to date, there is no available literature in this field. Moreover, OXT presents positive benefits on social cognition, emotional control, and bonding, which are known to help lessen the symptoms of psychosis [63], and it has immunomodulatory properties that may control the immune dysregulation in psychosis [64,65,66], even in FEP [67,68]. The increased OXT levels in the decidual cells of (FE-PW) might represent a compensatory mechanism that mitigates the symptoms of psychosis, according to the abovementioned effects of OXT.

Likewise, we demonstrated an important increase in both AVP and AVPR1A in both placental villi and decidual cells of FE-PW in comparison to healthy subjects. As far as we know, AVP expression in the placenta has not been recognized yet. Our results from RT-qPCR and IHC show that this hormone can also be produced by the human placenta or at least that it is possible to detect mRNA encoding AVP bound to neurophysin 2 as well as a protein product present in the placental tissue. Most studies have focused on AVP expression and function in the hypothalamus. However, previous studies have also found evidence of the expression of the neurophysin 2-vasopressin gene in extrahypothalamic tissues. For instance, Fuller et al. [69] reported the presence of AVP-neurophysin 2 mRNA in the ovary of Sprague-Dawley, Long-Evans, and Brattleboro rats, with an apparent molecular weight similar to that seen for the hypothalamus. Similarly, another study demonstrated that vasopressin was also expressed as three different transcripts in the rat testis [70], therefore supporting the extrahypothalamic expression of this hormone. Despite the fact that the human placenta also seems to express AVP-neurophysin 2, further studies are required to replicate these observations in the placental sample as well as studying possible autocrine or paracrine functions of this hormone in this tissue. In addition, there is limited knowledge about the role of AVP expression in the placenta under physiological and disease conditions. Sandgren et al. [71] observed that AVP secretion preceded pre-eclampsia symptoms and that this hormone had a potential role in the pathogenesis of this disease. This observation is also supported by previous studies, which showed that AVP administration seems to be sufficient to induce a pre-eclampsia-like phenotype in mouse models, and also it also promoted significant immunological changes in these patients [72,73]. Nevertheless, there are justifiable doubts as to whether the source of AVP comes exclusively from the brain and through which receptors AVP can exert its pathogenic role in the placental of women with pre-eclampsia. In this sense, previous studies have demonstrated the expression of AVPR1A in the placental tissue [26]. Altered epigenetic regulation of AVPR1a has been described in women with pre-eclampsia [30]. In sheep, AVPR1A expression increased in days 45 and 66 ± 1, during the maximal growth phase of the placenta. The authors proposed that increased levels of AVP in the bloodstream during heat stress in sheep could potentially trigger the activation of AVPR1a [26]. Thus, and as mentioned above, the study of circulating AVP in FE-PW could aid in establishing whether there is a possible relationship between circulating levels of AVP and its receptor. Additionally, these studies suggest a possible role of AVP and AVPR1a in the pathogenesis of obstetric complications like pre-eclampsia. However, their pathogenic implications in other obstetric and mental health complications during pregnancy such as FE-PW must be explored in future works.

Our study, however, has some important limitations that should be mentioned and addressed in future works. First, due to the low occurrence of FEP in pregnancy, the sample size of the women included in this study is limited. Additionally, we could not study either the plasma levels of OXT or AVP in the mothers and their newborns or the placental expression of two additional AVP receptors (AVPR1B and AVPR2). Moreover, we think that it would be really interesting to include the analysis of copeptin, another marker directly associated with AVP secretion whose role has been defined in a broad spectrum of complications, including psychiatric and obstetric disorders [25,74,75,76,77]. Thus, further efforts should be conducted to address these important limitations.

## 4. Patients and Methods

### 4.1. Study Design and Participants

A total of 42 pregnant women in their third trimester were the subjects of an observational, analytical, and prospective study. This consisted of FEP-pregnant women (FE-PW = 22) and healthy control pregnant women (HC-PW) n = 20. In the FE-PW group, the median gestational age was 40 (38–41) weeks, and the median age was 33.5 (21–42) years. The median gestational age for HC-PW was 40 (39–42) weeks, and the median age was 33.5 (25–39) years. Table 1 summarizes the sociodemographic and clinical characteristics of these patients.

Using the Structured Clinical Interview for DSM-5 (SCID-5) [78] and the Positive and Negative Syndrome Scale (PANSS) [79], a psychiatrist verified the diagnosis of FE-PW following the DSM-5 criteria. Being pregnant between the ages of 18 and 45 and fluency in Spanish to permit an accurate assessment were among the inclusion criteria. In contrast, exclusion criteria included: (1) having a current Axis-I mental disease that meets diagnostic criteria or having an intellectual handicap and (2) having a history of neurodevelopmental problems or having suffered a head injury that resulted in the loss of consciousness. Before enrollment, each subject received the necessary information, and they all gave their formally signed consent. The Clinical Research Ethics Committee of the Central University Hospital of the Defense University of Alcalá approved this work (37/17), and it was carried out by the guidelines for good clinical practice, the tenets of the Declaration of Helsinki (2013), the Oviedo Convention (1997), and the ethical principles of autonomy, beneficence, and non-maleficence.

### 4.2. Sample Collection and Processing

Following delivery, placental biopsies were taken from FE-PW and HC-PW. Five placental pieces were obtained for every sample and by using a scalpel, mixed cotyledons were included and divided into two different sterile tubes: one with RNAlater^®^ solution (Ambion; Thermo Fisher Scientific, Inc., Waltham, MA, USA) and another containing minimal essential medium (MEM; Thermo Fisher Scientific, Inc., Waltham, MA, USA) with 1% antibiotic/antimycotic (streptomycin, amphotericin B, and penicillin; Thermo Fisher Scientific, Inc.). Following that, placental samples were processed in a class II laminar flow hood (Telstar AV 30/70 Müller 220 V 50 MHz; Telstar; Azbil Corporation, Chiyoda-ku, Tokyo, Japan) under sterile conditions. To preserve samples for later processing and gene expression research, they were placed in 1 mL of RNAlater^®^ (−80 °C).

To eliminate erythrocytes, preserved MEM samples were washed and rehydrated five times in antibiotic-free MEM. A second scalpel was used to cut each sample into 2 cm pieces, which were then fixed in F13 (60% ethanol, 20% methanol, 13% distilled water, and 7% polyethylene glycol) by accepted procedures [80]. Following the development of paraffin-embedded samples using molds, sections of 5 µm thickness were cut using an HM 350 S rotary microtome (Thermo Fisher Scientific, Inc., Waltham, MA, USA) and collected after being placed in a hot water bath into glass slides treated with 10% polylysine. These samples were ultimately used in histological and immunohistochemical procedures.

### 4.3. Immunohistochemistry and Histological Visualization

The samples were initially deparaffinized using xylol and ethanol at progressively lower concentrations (100%, 96%, and 70%), and then they were hydrated in distilled water. Then, we performed immunohistochemistry by using an avidin–biotin complex and avidin peroxidase, which made it easier to identify antigen/antibody reactions by earlier methods [81]. PBS 1x was applied three times over the course of five minutes to placental tissues. After 30 min, non-specific binding sites were then blocked at room temperature (RT) with 3% bovine serum albumin (BSA) diluted in PBS. Samples were then incubated with the primary antibody for 90 min, followed by overnight incubation in PBS at 4 °C with 3% BSA Blocker (cat. no. 37525; Thermo Fisher Scientific, Inc., Waltham, MA, USA). The next day, placental samples were incubated for 90 min at RT with biotin-conjugated secondary antibodies that had previously been diluted in PBS (Table 2). Then, the avidin–peroxidase conjugate ExtrAvidin^®^-Peroxidase (Sigma-Aldrich; Merck KGaA, San Luis, MO, USA) was added for one hour at RT (1:200 dilution in PBS). Finally, a chromogenic diaminobenzidine (DAB) substrate kit (cat. no. SK-4100; Maravai LifeSciences, San Diego, CA, USA), prepared just before exposure (5 mL distilled water; four drops of DAB; two drops of hydrogen peroxide and two drops of buffer) was employed to determine the protein expression level.

The signal can be detected as brown staining by using the peroxidase chromogenic substrate for 15 min at RT. Negative controls were assigned for each protein (without including the primary antibody), and Carazzi hematoxylin was employed for 15 min to assess contrast in all tissues in place of the primary antibody’s incubation. The protocol guidelines (Table 2) provide specifics on the antibodies used in our study.

Eventually, a Zeiss Axiophot optical microscope (Carl Zeiss, Oberkochen, Germany) was employed for histological analysis. Five distinct sections and ten fields were randomly investigated by two independent histologists for each placental sample. Following the immunoreactive score (IRS) established in previous works, immunohistochemical expression was deemed positive when the stained mean area in the investigated sample was 5% [82]. Immunostaining can be categorized using this method on the following scales: 0–1, minimal staining (25%), 2, moderate staining (25–65%), and 3, intense staining (65–100%).

### 4.4. Gene Expression Study

As stated in earlier publications [83], the guanidinium thiocyanate-phenol-chloroform technique was used to enable RNA extraction. Analysis of the mRNA expression levels of particular genes is made easier by this technique.

Reverse transcription was used to create complementary DNA (cDNA) from 50 ng/µL of RNA samples. RNA denaturation was facilitated by mixing 4 µL of each sample with 4 µL of 0.25 µg/µL oligo-dT solution (Thermo Fisher Scientific, Inc., Waltham, MA, USA) before placing the mixture at 65 °C for 10 min in a dry bath (AccuBlock, Labnet International Inc., NJ, USA). Following that, samples were placed on ice with 10 µL of an RT mix that contained the following items (All from Thermo Fisher Scientific, Inc., Waltham, MA, USA): 2.8 µL of First Strand Buffer 5× composed by 250 mM Tris-HCl and pH 8.3; 375 mM KCl:15 mM MgCl_2_ (Thermo Fisher Scientific, Inc., Waltham, MA, USA); 1 µL of retro transcriptase enzyme (all from Thermo Fisher Scientific, Inc., Waltham, MA, USA); 2 µL of 10 mM deoxyribonucleotide triphosphate; 2 µL of 0.1 M dithiothreitol; 1.7 µL of DNase and RNase free water; and 0.5 µL of RNase inhibitor (RNase Out).

Reverse transcription was completed using the G-Storm GS1 thermocycler (G-Storm Ltd.). To promote cDNA synthesis, the samples were incubated for 1 h and 15 min at 37 °C. The retrotranscriptase was then denaturized by increasing the temperature to 70 °C and holding it there for 15 min. The temperature then gradually dropped to 4 °C. Negative RT, in which the M-MLV reverse transcriptase was swapped out for DNase- and RNase-free water, which was carried out to guarantee that there was no genomic DNA contamination in the RNA samples. The cDNA made at RT was diluted in water devoid of DNase and RNase (1:20) and stored at −20 °C until use.

Table 3 lists the specific primers for each gene. The Primer-BLAST and AutoDimer online tools were used for the design [84,85]. To standardize our findings, we employed the constitutively expressed gene TATA-box binding protein (TBP) as a control [86]. Relative mRNA concentrations were used to express gene expression units. The relative standard curve method was used to conduct RT-qPCR using a StepOnePlus^TM^ System (Applied Biosystems; Thermo Fisher Scientific, Inc.). The following occurred as a result: forward and reverse primers were mixed with 5 µL of material, 10 µL of iQ^TM^ SYBR^®^ Green Supermix (Bio-Rad Laboratories, Inc.), and 3 µL of DNase- and RNase-free water, and they were mixed at a ratio of 1:20 before being added to a MicroAmp^®^ 96-well plate (Applied Biosystems; Thermo Fisher Scientific, Inc., Waltham, MA, USA). The following conditions were used during the 40–45 cycles of thermocycling: initial denaturation for 10 min at 95 °C, denaturation for 15 s at 95 °C, annealing at various temperatures according to the melting temperature of each primer pair for 30 s, and elongation at 72 °C for one minute. Then, a dissociation curve was developed for 15 s at 95 °C, 1 min at 60 °C, 15 s at 95 °C, and 15 s at 60 °C. After each repeat cycle (amplification) and at various points along the dissociation curve, fluorescence detection was carried out. The information gathered from the genes was incorporated into a standard curve that was created by serially diluting a variety of samples, which were included in each plate by the constitutive expression of TBP (according to the manufacturer’s guidelines). All placenta tissue samples underwent this RT-qPCR twice to ensure consistent results.

### 4.5. Statistical Analysis

GraphPad Prism^®^ v6.0 (GraphPad, Inc., San Diego, CA, USA) was used for the statistical analyses, and the Mann–Whitney U test was used to compare the two groups. The median and interquartile range (IQR) were used to express the results. The thresholds for significance were set at *p* < 0.05 (*), *p* < 0.01 (**), and *p* < 0.001 (***).

## 5. Conclusions

**Figure 5:**
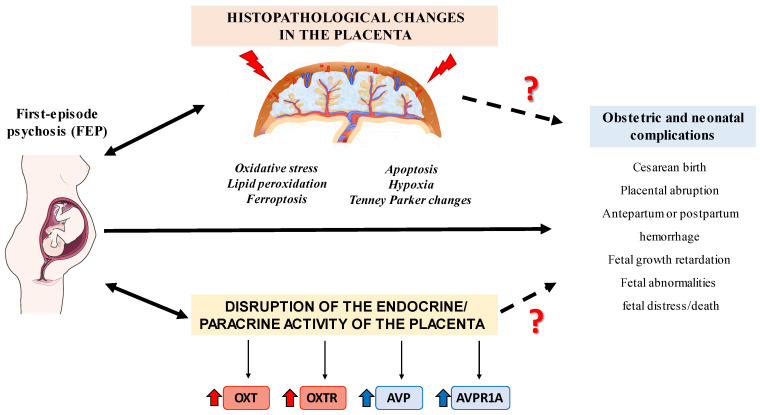
Graphical summary of the study. The first-episode psychosis (FEP) during pregnancy increases susceptibility to a number of obstetric and neonatal complications. In addition, FEP has been associated with several histopathological changes in the placenta and increased tissue expression of oxytocin (OXT), arginine vasopressin (AVP), and their receptors (OXTR and AVPR1A, respectively) in this organ. Future studies should deepen our understanding of the association between FEP in pregnancy and placental alterations with the obstetric and neonatal complications observed in these patients.

In this study, we found an increased expression of OXT/OXTR and AVP/AVPR1A in the placental tissue of FE-PW compared to HC-PW. These findings suggest a potential association between altered levels of these hormones and their receptors in the placenta and the occurrence of psychosis during pregnancy, which could contribute to adverse obstetric and neonatal outcomes (Figure 5). However, further research is necessary to fully understand the implications and consequences of these observed alterations.

## Figures and Tables

**Table 1:** Clinical and demographic traits of pregnant women who experienced their first psychotic episode and healthy controls. FE-PW = first psychotic episode during pregnancy, HC-PW = healthy control pregnant women.

	FE-PW (n = 22)	HC-PW (n = 20)
Median age (IQR), years	33.5 (21–42)	33.5 (25–39)
Median gestational age (IQR), weeks	40 (38–41)	40 (39–42)
C-section delivery, n (%)	3 (13.6)	2 (10.0)
Previous pregnancies, n (%)	8 (36.4)	9 (45.0)
Previous abortions, n (%)	1 (4.5)	2 (10.0)
Regular menstrual cycles, n (%)	17 (77.3)	16 (80.0)
PANSS mean (SD)	Positive 18.8 (6.3)	-----
	Negative 25.7 (7.9)	

**Table 2:** Primary and secondary antibodies and their dilutions.

Antigen	Species	Dilution	Provider	Protocol Specifications
Anti-neurophysin 1/NP-OXT antibody	Rabbit Polyclonal	1:250	Abcam (ab2078)	EDTA pH = 9, before incubation with blocking solution
OXTR	Rabbit Polyclonal	1:500	Thermofisher (PA5-34066)	EDTA pH = 9, before incubation with blocking solution
Anti-Neurophysin 2/NP-AVP Antibody	Mouse Monoclonal	1:350	Sigma-Aldrich (MABN845)	10 mM sodium citrate pH = 6, before incubation with blocking solution
AVPR1a	Rabbit Polyclonal	1:500	LSBio (LS-A3831)	100% triton, 0.1% in PBS for 10 min, before incubation with blocking solution
IgG (Rabbit)	Mouse	1:1000	Sigma-Aldrich (RG96/B5283)	------
IgG (Mouse)	Goat	1:300	Sigma-Aldrich (F2012/045K6072)	------

**Table 3:** Primers used for RT-qPCR: sequences and binding temperatures (Temp).

GENE	SEQUENCE Fwd (5′→3′)	SEQUENCE Rev (5′→3′)	Temp
TBP	TGCACAGGAGCCAAGAGTGAA	CACATCACAGCTCCCCACCA	60 °C
Human preprooxytocin-neurophysin I gene	TAAAAAGGCCAGGCCGAGAG	TCTTCCAGTCCCACAATGCC	57 °C
OXTR	TCCTGTACCCATCCAGCGA	TCCGCAGGCGAACCTAAAG	60 °C
Human prepro-8-arginine-vasopressin-neurophysin II gene	GCTGCCAGGAGGAGAACTAC	GAGACTGAGACAGACGCGAG	58 °C
AVPR1a	TGGGCGCCTTTCTTCATCAT	AGGGTTTTCCGATTCGGTCC	61 °C

## Data Availability

The data used to support the findings of the present study are available from the corresponding author upon request.
